# Footwear and insole design parameters to prevent occurrence and recurrence of neuropathic plantar forefoot ulcers in patients with diabetes: a series of N-of-1 trial study protocol

**DOI:** 10.1186/s13063-022-06968-5

**Published:** 2022-12-16

**Authors:** Sayed Ahmed, Paul Butterworth, Alex Barwick, Anita Sharma, Md Zobaer Hasan, Susan Nancarrow

**Affiliations:** 1 School of Health and Human Sciences, Southern Cross University, Billinga, Queensland 4225 Australia; 2 Department of Geriatric Medicine, Nepean Hospital, Penrith, New South Wales Australia; 3 Nepean Clinical School, Faculty of Medicine and Health, University of Sydney, Camperdown, New South Wales Australia; 4 School of Science, Monash University Malaysia, Jalan Lagoon Selatan, 47500 Bandar Sunway, Selangor Darul Ehsan Malaysia; 5 General Educational Development, Daffodil International University, Daffodil Smart City, Ashulia, Dhaka, Bangladesh

**Keywords:** Diabetes, Polyneuropathy, Foot ulcer, Footwear, Insoles

## Abstract

**Background:**

Foot complications occur in conjunction with poorly controlled diabetes. Plantar forefoot ulceration contributes to partial amputation in unstable diabetics, and the risk increases with concomitant neuropathy. Reducing peak plantar forefoot pressure reduces ulcer occurrence and recurrence. Footwear and insoles are used to offload the neuropathic foot, but the success of offloading is dependent on patient adherence. This study aims to determine which design and modification features of footwear and insoles improve forefoot plantar pressure offloading and adherence in people with diabetes and neuropathy.

**Methods:**

This study, involving a series of N-of-1 trials, included 21 participants who had a history of neuropathic plantar forefoot ulcers. Participants were recruited from two public hospitals and one private podiatry clinic in Sydney, New South Wales, Australia. This trial is non-randomised and unblinded. Participants will be recruited from three sites, including two high-risk foot services and a private podiatry clinic in Sydney, Australia. Mobilemat™ and F-Scan® plantar pressure mapping systems by TekScan® (Boston, USA) will be used to measure barefoot and in-shoe plantar pressures. Participants’ self-reports will be used to quantify the wearing period over a certain period of between 2 and 4 weeks during the trial. Participant preference toward footwear, insole design and quality-of-life-related information will be collected and analysed. The descriptive and inferential statistical analyses will be performed using IBM SPSS Statistics (version 27). And the software NVivo (version 12) will be utilised for the qualitative data analysis.

**Discussion:**

This is the first trial assessing footwear and insole interventions in people with diabetes by using a series of N-of-1 trials. Reporting self-declared wearing periods and participants’ preferences on footwear style and aesthetics are the important approaches for this trial. Patient-centric device designs are the key to therapeutic outcomes, and this study is designed with that strategy in mind.

**Trial registration:**

Australian New Zealand Clinical Trials Registry (ANZCTR) ACTRN12620000699965p. Registered on June 23, 2020

## Administrative information

**Table:** 

Title	Footwear and insole design parameters to prevent occurrence and recurrence of neuropathic plantar forefoot ulcers in patients with diabetes; - A series of N-of-1 trials.
Trial registration	This trial has been registered with the Australian New Zealand Clinical Trials Registry (ANZCTR), and the registration number is ACTRN12620000699965p. Date: June 23, 2020
Protocol version	December 2021, Version 1.9
Funding	As the footwear and insoles are part of the standard treatment for each participant, the funding will be provided by the funding source to which each participant has access. This includes HealthShare Enable NSW [1], private health funds, aged care packages, Closing the Gap or self-funded by participants based on their eligibility criteria. Foot Balance Technology (owned by the chief investigator) will provide support for the F-scan and MatScan systems and relevant sensors. There will be no cost to participants for the sensors.
Author Details	Sayed Ahmed^1^ Dr Paul Butterworth^1^ Dr Alex Barwick^1^ Dr Anita Sharma^2,3^ Dr Md Zobaer Hasan^4,5^ Professor Susan Nancarrow^1^ ^1^School of Health and Human Sciences, Southern Cross University, Queensland, 4225, Australia ^2^Department of Geriatric Medicine, Nepean Hospital, Penrith, New South Wales, Australia ^3^Nepean Clinical School, Faculty of Medicine and Health, University of Sydney, New South Wales, Australia ^4^School of Science, Monash University Malaysia, Jalan Lagoon Selatan, 47500 Bandar Sunway, Selangor Darul Ehsan, Malaysia ^5^General Educational Development, Daffodil International University, Daffodil Smart City, Ashulia, Dhaka, Bangladesh.
Name and contact information for the trial sponsor	Sayed Ahmed, Director, Foot Balance Technology Pty Ltd Email: sayed@footbalancetech.com.au
Role of sponsor	Foot Balance Technology will support the F-scan and MatScan system (including hardware and software) and relevant sensors. There will be no cost to participants for the sensors.

## Background

Foot ulcers are a common consequence of diabetes due to the development of peripheral neuropathy, peripheral vascular disease, limited joint mobility and foot deformity [2–7]. Nearly 34% of people with diabetes will develop a foot ulcer in their lifetime [8]. This can lead to infection and amputation; diabetes is the main reason for non-traumatic amputation [9, 10]. Previous foot ulceration or amputation is a risk for future amputation [2, 4, 6, 11]. Additional risk factors include a higher body mass index (BMI) and structural foot deformities [3–5, 7], such as hammertoes and hallux valgus [12, 13].

Diabetic peripheral neuropathy (DPN) is a risk factor for the development of ulceration [14]. Over 30% of persons with diabetes will develop DPN [15], the incidence increasing with age [16, 17]. DPN can affect the autonomic, sensory and motor nervous systems. Sensory neuropathy interrupts the protective feedback mechanism of touch and pain [18]. Motor neuropathy results in compromised muscle innervation, reduction in strength and, combined with limited joint mobility, the development of foot deformities. These deformities may lead to an increase in plantar foot pressures, particularly in the forefoot [19–22]. Autonomic neuropathy leads to diminished sweating and changes to skin perfusion, leading to dry skin and hyperkeratosis. As skin integrity is compromised, patients are more susceptible to trauma which may predispose a diabetic foot ulcer [22–25].

Neuropathic foot ulcers in persons with diabetes occur mostly at the plantar forefoot [12, 26, 27] and correspond to areas of peak plantar pressure (PPP) [28]. Bennetts et al. [29] demonstrated that most peak pressure areas are located in the forefoot regions in this population. A limited range of motion at the forefoot joints is also likely to contribute to the increased PPP observed in this region [30]. For this reason, plantar pressure mapping is used to guide footwear and insole manufacture and judge their effectiveness [31].

Reducing plantar pressures is considered a key factor for wound healing and the prevention of ulcer recurrence [32, 33]. Footwear and insoles are important treatment modalities for offloading these pressures [34, 35]. The desired offloading threshold should be <200 kPa to ensure ulcer-free survival at the forefoot [36]. Some studies also recommended that a pressure relief of 25–30% compared with the baseline be effective [31, 37]. The evidence for effective design characteristics of footwear and insole that can reduce plantar pressure is limited in the literature [38], and further exploration of the various design and modifications of footwear and insole can bridge the gap in the literature [39, 40].

### The rationale for performing the study

There is no existing, evidence-based recommendation for overall footwear and insole design that includes all technical specifications to offload the diabetes-related and neuropathic foot [38–40]. Several studies have suggested components of footwear design, such as the rocker sole profile, as the preferred design feature to offload PPP at the forefoot [31, 41–45]. Arts et al. [46] in the Netherlands and Rizzo et al. [43] in Italy conducted studies to test the effect of footwear design suggested by the consensus-based algorithm proposed by Dahmen et al. [47]. Both studies found that the footwear and insole design is effective in offloading the neuropathic diabetic foot. However, Arts and colleagues [46] found that the algorithm is not as effective for footwear specifications when offloading plantar pressure at the metatarsal heads is required.

Several studies [31, 43, 45, 48–50] have explored patient satisfaction and adherence to wearing footwear and insoles. Patient adherence to wearing therapeutic footwear is important to ensure improved offloading and ulcer prevention [43, 45, 48].

### Research aim

The aim of this study is to identify clinically relevant footwear and insole design and modification parameters that effectively offload forefoot PPP in the neuropathic feet of people with diabetes and increase adherence.

### Objectives

The research questions are:

- What factors and parameters need to be considered when prescribing footwear and insoles for people at risk of neuropathic forefoot plantar ulcer occurrence and recurrence?
- How can participants’ preferences be incorporated into footwear and insole design to increase the adherence to prescribed footwear in people with diabetes and neuropathy who are at risk of plantar forefoot ulcer occurrence and recurrence?

### Expected outcomes

The overall goal of this intervention is the prevention of ulceration; however, ulceration is an undesirable outcome for participants, so a proxy measure, which is a reduction of plantar pressure using footwear, is used as the primary outcome measure instead.

The expected outcome of the study is an algorithm or best practice recommendations that can be personalised to prescribe footwear and insole design and modifications based on individual pathologies, comorbidities and lifestyles in people with diabetes and peripheral neuropathy who are at risk of plantar forefoot ulceration. This algorithm or best practice recommendation is expected to provide a clear guideline for referrers, patients, prescribers and technicians to design or modify footwear and insole to ensure adequate and effective offloading of the forefoot to prevent the occurrence and recurrence of plantar neuropathic ulcers in people with diabetes.

The recommendations are expected to influence better optimise health service models and health fund’s resource allocation to maximise the impacts.

## Methods

### Trial design

**Fig. 1:**
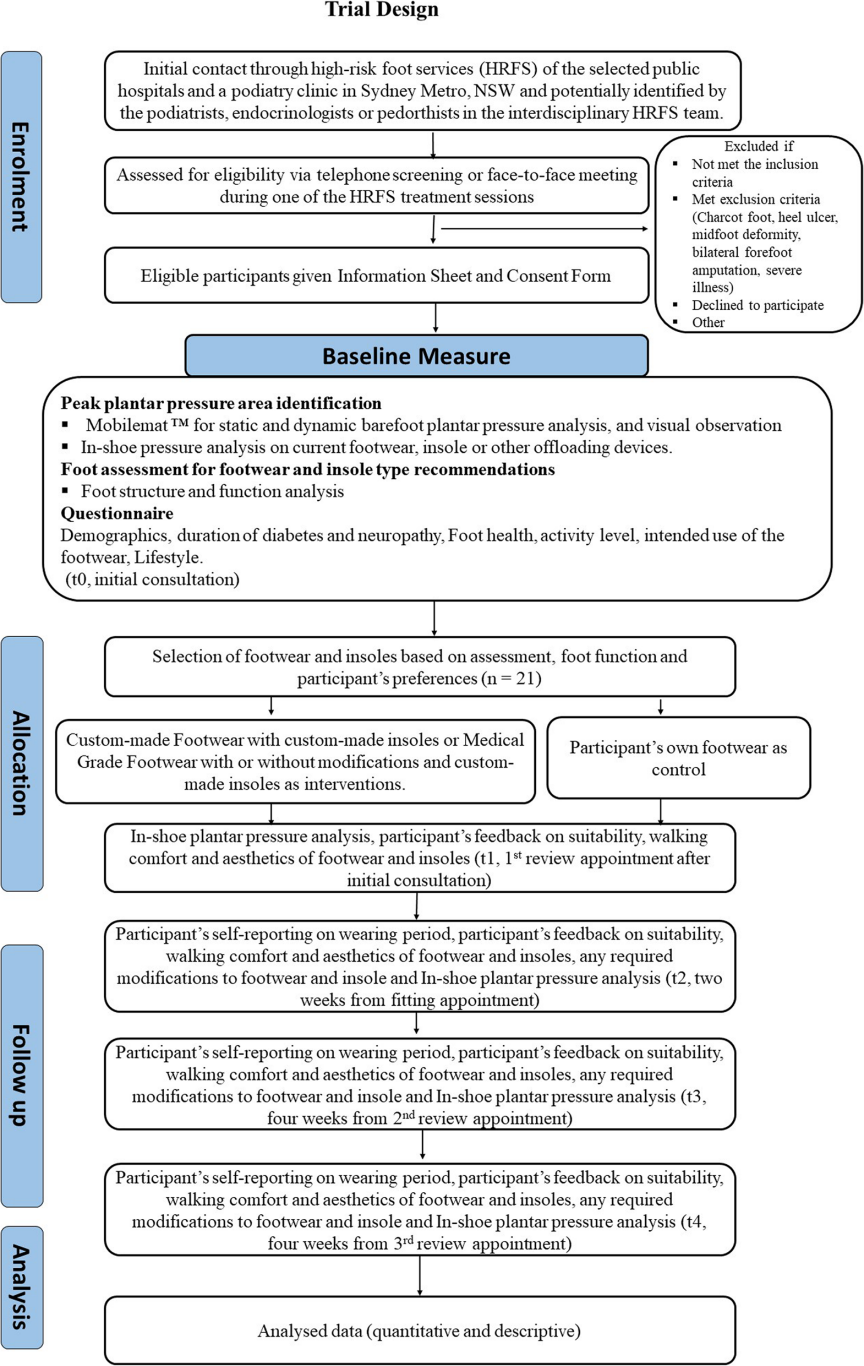
Trial design flow for the N-of-1 trial

The trial design is shown in the flowchart (Fig. 1).

### Methodological approach

The study will be comprised of a single patient or N-of-1 trial.

N-of-1 trials are usually randomised, often double-blind, and involve multiple crossover comparisons of an intervention and a control treatment [51, 52]. Oxford Centre for Evidence-Based Medicine has recommended this trial as level 1 evidence for treatment decisions [53].

N-of-1 trials provide a technique to guide evidence-based treatment decisions for an individual patient. They use common methodological components of large clinical trials to measure treatment effectiveness in a single patient. They are a practical alternative when circumstances do not allow for large-scale trials, such as rare diseases or comorbid conditions, or when participants use concurrent therapies [54]. However, the findings from these trials can be used to inform the development of algorithms to guide complex treatments for other patients. In this case, the complexity of the intervention (footwear and insole design) and the individually tailored nature of the intervention lend itself to an N-of-1 trial. This is also a more cost-effective approach than traditional phase three clinical trials [55].

Methodologically robust N-of-1 trials objectively assess the effectiveness of treatments within individual participants. Aggregation of multiple cycles identically conducted N-of-1 trials yields a population estimate of effect, which approximates the similar effect derived from other RCT designs. Trial participants contribute data for both intervention and control treatments creating matched data sets while using generally smaller sample sizes than conventional RCTs [51].

Single-patient or N-of-1 trials are commonly used for personalising the treatment options when participants have a chronic condition [56]. Recent studies suggest that N-of-1 trials are effective tools for improving therapeutic precision, and they are widely accepted by patients and clinicians as an effective modality because they are patient-centred [56, 57]. They also have proven value in guiding a more effective prescription [55, 57, 58]. In the era of ‘personalised medicine’, they are becoming more popular. It is increasingly clear that ‘one size does not fit all’, particularly in complex interventions like diabetic footwear design and modifications.

In this proposed study, participants will have an initial assessment with the principal investigator, having been referred from the high-risk foot service. The initial assessment will include the selection of appropriate footwear, measuring, casting and 3D scanning of feet and technical specification of footwear and insole that reflects participant’s preferences. Barefoot static and dynamic pressure and in-shoe pressure measurements will be carried out. Current (standard) footwear, which may be a regular retail shoe, orthopaedic footwear, post-op shoes or cam-walkers or moon boots with or without insoles (custom or prefab), will be used as the control arm for the trial.

The second assessment will be done once the footwear and insole are ready for fitting (generally within 4 weeks after the initial assessment and measurement). In-shoe pressure analysis will be carried out in the new footwear and insole, which may undergo minor or major modification to achieve desired offloading efficacy. Participants will be assessed for walking comfort and satisfaction with their new footwear. Scores will be recorded on a Likert scale [59]. A third consultation will be arranged 2 weeks later. New footwear-wearing instructions will be provided to the participants, including contact details for an emergency or experiencing any adverse effect from the new footwear.

In the third assessment, the participant’s feet will be assessed for any redness or rubbing and any discomfort from the footwear. The participant’s self-reported wearing period data will be collected and analysed for wear patterns and frequency. The footwear will be assessed for any unusual wear marks or pressure points. Necessary adjustments will be made, and the fourth review session will be booked for a monthly visit.

During the fourth assessment, the participant’s feet and footwear will be assessed again. Participants’ satisfaction with the footwear will be recorded and compared with the participant’s self-reported wearing period data for the previous month. A similar process will follow, and another monthly appointment will be made with the researcher.

In the fifth assessment, a similar process will follow as the previous review, and this is the endpoint of the study. Participants will be asked about the walking comfort and likeliness of footwear and suitability for the intended application. Overall feedback and any comments from the participants on the footwear will be recorded. Any repair or adjustments to the footwear and insoles will be carried out, and they will be given contact details for any future repair and follow-up reviews.

Double-blinding is not feasible in this study design, given the nature of the intervention and secondary outcomes. Blinding is recommended in N-of-1 trials, but it is not mandatory [52]. In this proposed study design, participants will need to be aware of the prescribed footwear in order to provide feedback on acceptance. Features of the footwear and insoles may be easily identifiable by clinicians in the team. However, the statistician will remain blinded, and this study design can be termed a single-blind trial [60, 61].

### Reporting standard

The quality of any study is dependent on the reporting standard of the study. To increase validity and acceptability, this N-of-1 trial will report data as per CONSORT extension for reporting N-of-1 trials (CENT) 2015 Statement [54].

### The rationale for the N-of-1 trial

Patient adherence is key for successful offloading initiatives of the neuropathic diabetic foot. Footwear is an integral part of clothing. Patient preferences play a vital role in footwear usage and adherence to recommendations. Therefore, a patient-centred study design that can recommend a precise prescription on personalised therapy/devices is important. The N-of-1 trial design is unique in that it allows a focused assessment of patient preferences and circumstances. This is also beneficial for personalised treatment decisions for patients with chronic conditions [56]. There is a direct clinical application in individualising each participant’s treatment with outcomes generalisable to a broader patient population [56].

The effectiveness of offloading will be measured using the in-shoe plantar pressure analysis system. Using in-shoe plantar pressure measurements and [62] analysis is the gold standard in footwear-based pressure reduction studies [31, 63].

This trial methodology is preferable to participants as they feel more involved in treatment decisions and see changes being made in response to their feedback [51].

### Rationale for control

The primary outcome measure is the reduction of forefoot peak plantar pressure. The control will be participants’ existing, usual footwear and insoles in the form of regular, orthopaedic or post-op footwear. This will form the baseline data of in-shoe plantar pressure measurements to be compared against the new footwear and insole to evaluate offloading efficacy. Hence, the choice of a control arm is essential, but in this case, the patient is their own control.

### Study setting

Proposed physical sites are high-risk foot services (HRFSs) of the following hospitals and a private podiatry clinic:

1. Offloading clinic of Nepean Hospital, Nepean Blue Mountains Local Health District
2. St Vincent’s Hospital Sydney, St Vincent’s and Mater Health Network
3. Western Sydney Podiatry, Penrith, NSW 2750

### Eligibility criteria

#### Inclusion criteria

Participants will be adults (≥18 years) with type 1 or type 2 diabetes, peripheral neuropathy and recently healed plantar forefoot ulcer. Participants must have at least one or more forefoot deformities such as claw/hammer toes, crossover toes, hallux valgus, hallux amputation, limited joint mobility, pes planus or pes cavus and bony prominences at metatarsal heads. Participants will have required a referral for orthopaedic footwear (either custom-made or prefabricated medical-grade footwear with or without modification) and custom-made insoles and have adequate English communication skills to comprehend the study procedures and provide informed consent.

#### Exclusion criteria

Exclusion criteria will be bilateral amputation (proximal to the trans-metatarsal joint), active or inactive Charcot foot, healed heel ulcers, midfoot deformities, use of walking aid for offloading the foot or severe illness, such that the participant may not survive the study period. Details are provided in the trial flow chart.

### Participant’s consent process

The participant will meet with the investigator prior to completing informed consent. The investigator will explain the rationale for the study, what participation will involve, follow-up requirements if applicable and any side effects or risks. Questions from the participant will be encouraged. The participant information sheet (PIS) will be provided and discussed with the participant. The participant may wish to consider their decision or discuss it with other parties, and in that case, another visit with the investigator can be arranged. The participants will be advised that they can withdraw from the study at any time without explanation or prejudice to future care. If willing, the participant will be asked to sign the informed consent form. The principal investigator for each site will sign the PIS after the participant has signed.

## Interventions

### Choice of comparisons

In this trial, participants’ existing footwear, insoles or offloading devices are the comparators. Participant’s current (standard) footwear, which may be regular retail shoes, orthopaedic footwear, post-op shoes or cam-walkers or moon boots with or without insoles (custom-made or prefabricated), will be used as the control arm for the trial.

### Intervention description

Footwear and insole are the interventions in this trial which are personalised and can be modified further to improve PPP offloading and increase adherence [39, 40]. Participants are required to select a preferred style of footwear and report on the activities for which they intend to wear the footwear. The choices of footwear are either prefabricated orthopaedic footwear with or without modification and fully custom-made orthopaedic footwear, which is made from individual foot measurements and 3D scans of the foot and leg. The insoles will be designed in response to foot assessment data, a foam impression box and a 3D scan of the individual foot. The insoles will be manufactured using conventional manufacturing or 3D printing method. Participants will be provided with instructions for wearing the footwear and insoles. The footwear and insole suitability for the participants will be carefully checked at the t1 appointment by the researcher and also by the referring podiatrist, which is part of the regular clinical protocol. This process will be continued during each visit to ensure they are suitable for the participant. The modification of footwear, insole and in-shoe plantar pressure measuring will continue until an acceptable pressure reduction is achieved and the participant is satisfied with them. In the unlikely event that an adverse effect is observed from the provided footwear and insole, the participant will be asked to stop using them until a further assessment is conducted by the researcher and the treating multidisciplinary team.

### Strategies to improve adherence to interventions

The interventions in this trial are therapeutic footwear and insole that are personalised and can be modified further when needed. These are wearable items of clothing for the participant. Participants’ preferences and the suitability of use will be considered in the selection of footwear interventions. There will be regular follow-up with participants, and participants’ self-reported data on adherence and satisfaction with the footwear and insole will be collected at each appointment. Participants will be given the motivation to adhere to the therapies by the researcher and other research team members, including explanations of the benefits of the devices and the adherence.

### Provisions for post-trial care

These participants are at high risk of foot re-ulceration and are advised to be under regular podiatry care either at the community health clinics or their private podiatry clinics. They are also advised to visit the nearest high-risk foot services for any unlikely incident with their feet, such as an active wound or infection, with a referral from their GP or via the emergency department of the respective hospital. Participants will be advised to undergo twelve-weekly reviews with their pedorthists to ensure the footwear and insoles are repaired and optimally maintained.

### Outcomes

The primary outcome is a reduction of PPP at the accepted level according to the protocol (<200kPa or 30% reduction from the baseline/control) [64]. The outcome will be measured by using an in-shoe pressure analysis (F-Scan® system by Tekscan® [65]).

The participants will also undergo in-shoe plantar pressure measurements at each fitting and review appointment, where they need to walk up to 12 m at a self-selected pace that represents their regular pace of walking and is consistent during each measurement. F-Scan® sensors will be calibrated at ‘Walk’ calibration, and the body weight of the participant will be recorded each time during the analysis.

The secondary outcome is adherence (recorded from the participant’s self-report on the wearing period) and participant’s satisfaction with the provided footwear and insoles (in terms of walking convenience and aesthetics, to be measured by using a Likert scale [59]).

### Participant timeline

This project will last between 3 and 4 months, including the initial assessment, fitting of footwear and insoles and then at least three reviews outlined in the trial design flowchart in Fig. 1. The details of the timelines are t0, initial consultation; t1, 1st fitting appointment after the initial consultation; t2, 2-week review from fitting appointment; t3, 4 weeks from the 2nd review appointment; and t4, 4-week review from the 3rd review appointment.

### Sample size

Twenty-one participants [51] from the HRFSs of two major public hospitals and their affiliated community clinics (Nepean Hospital, St Vincent’s Hospital Sydney) and a private podiatry clinic (Western Sydney Podiatry) in Sydney will be recruited for the study. A sample size calculation is not possible due to the non-existence of a well-validated Quality of Life Scale (QOLS) for the target population. The QOLS measures an individual’s satisfaction, perceptions of control, involvement, commitment and work-life balance regarding an individual’s personal perception. Previous studies [51, 66–68] utilising the N-of-1 methodology have recruited between 10 and 25 participants.

### Recruitment

Participants will be recruited from the aforementioned HRFSs and the private podiatry clinic. Potential participants will be identified by interdisciplinary team members of the HRFSs, including endocrinologists, pedorthists and podiatrists. Participants will then be invited to participate.

### Accounting for potential bias, confounding factors and missing information

There is a potential risk of biasing the results for the researcher also being the treating clinician. However, the statistician will remain blinded to the condition of the participant. The participant will also remain de-identified to the statistician, including the footwear and insole design features prescribed to that specific participant. The study is non-randomised, and any missing data will be handled through ‘as treated’ analysis. The in-shoe plantar pressure measurement will be done by using the F-Scan® system by Tekscan® [65] and the F-Scan® research software 7.0. The software generates the pressure analysis report without the clinician’s intervention. The report is based on sensors calibration data and actual interaction of pressure between the foot, insole and footwear. Thus, the report remains independent of external influence to give a true reflection of the offloading efficacy of the footwear and insole’s design and subsequent modifications. Participant’s self-report on perceived clinical outcomes regarding plantar pressure offloading (<200kPa or 30% reduction from the baseline/control), suitability of the footwear and insoles and the review feedback from the treating podiatrists in the high-risk foot services will also reduce the risk of bias.

### Data collection

Each participant’s medical history, details of their foot assessment and comorbidities will be recorded in Qualtrics software [69], either directly into Qualtrics or from a paper-based case report form (CRF) to Qualtrics. This information will be obtained from the treating high-risk foot service with the participants’ consent. Participants’ preferences and adherence-related information will also be recorded in the same software for analysis following a similar data entry process. Plantar pressure data and shoe wear period data will be collected at each appointment with the researcher. Persons will be de-identified/anonymised before sending the data to the statistician.

### Data management

Each participant will receive a study enrolment number, which will be used on an electronic spreadsheet. This way, participants can be re-identified by utilising the study enrolment number when further data collection or clarification is required. Once collated, data will be non-identifiable.

Data management and storage will be maintained in line with the National Health and Medical Research Council’s (NHMRC) ethics requirements [70]. Any hard copy, such as the participant’s signed consent form, CRF’s will be scanned and stored in the form of an electronic copy, and hard copies will be disposed of in a locked confidentiality bin. Electronic data will be stored on a password-protected computer with an up-to-date version of Trendmicro Maximum Security antivirus software. For each site, a Research Data Management Plan (RDMP) form will be maintained. Initial data will be stored at Southern Cross University (SCU)-approved data storage system for analysis purposes, and towards the end of the project, the data will be stored at SCU-approved and recommended data repositories.

Data will be archived for a period of 15 years after study completion, and this is the maximum recommended period of data archive period by NHMRC ethics requirements guidelines. This period will allow for conducting any follow-up study if the opportunity arises. After that period, electronic data will be securely erased.

### Data analysis

Both descriptive and inferential statistical techniques will be used in this research. In the descriptive analysis, patient characteristics, as well as adherence/wearing time based on the participant’s satisfaction with the footwear and insoles, will be summarised. Under the statistical inference, a paired sample *t*-test will be used to compare the significant difference in plantar pressure between the custom-made footwear and baseline or control footwear. Two independent samples *t*-tests will be applied to compare patient characteristics between low- and high-adherence groups as well as participant’s compliance data and the clinician’s recommended data. In addition, the independent samples *t*-test will be used to compare the demographics and clinical features of the patients admitted to public and private clinics. Furthermore, the Wilcoxon signed-rank test would be used to compare adherence and wearing time between both follow-up moments and baseline. The descriptive and inferential statistical analyses will be performed using IBM SPSS Statistics (version 27). And the statistical significance will be set at *p*<0.05 with a confidence limit at 95% in a two-tailed fashion. For the qualitative data analysis, a thematic analysis will be used, and the software NVivo (version 12) will be utilised for that purpose.

### Matching and sampling strategies

The participants are their own controls, so all data (plantar pressure data) will be matched within the participants for the intervention and control arms of the study.

### Plans to give access to the full protocol, participant-level data and statistical code

The full study protocol, de-identified participant-level data and statistical code can be available by contacting the author.

## Oversight and monitoring

### Research governance and ethics

This multisite trial is administered by SCU and NBMLHD HREC and the RGOs of St Vincent’s Hospital Sydney and Nepean Hospital. The ethics have been approved by NBMLHD and the SCU HREC. The approval numbers are 2020/ETH02250 and 2020/093, respectively. The trial is conducted in accordance with the NHMRC guideline [70].

Written informed consent is obtained from each participant by one of the research teams of the respective site at the time of initial assessment and baseline information collection. A copy of the consent form can be available by contacting the corresponding author.

### Trial management and data monitoring committee

The named authors in this article will be part of the trial management committee, which will also include each site-specific investigator and the collaborators reported in the study protocol. The chief investigator will ensure that every co-investigator is actively participating to ensure the participant’s safety and the trial quality is maintained. The lead chief investigator conducts the audit and reviews. Every principal coordinating investigator named in the project protocol for each site will be taking responsibility for the data monitoring.

### Adverse event reporting and harms

Risk in this study relates to the use of new footwear and insoles for the participant.

Participants may face the following risks:

- Risk of fall or feeling unbalanced with wearing new footwear and insole to start.

Risk mitigation: Participants will be assessed carefully for any potential risk of falls and heel height, and the rocker profile will be adjusted accordingly in the footwear to mitigate the risk of falls or improve balance. At the initial fitting stage, the principal investigator will walk with the participant and will show the appropriate way of walking in the new devices.

b. Risk of developing a blister or pressure mark either on the plantar or dorsal surface of the foot and leg. This can be due to changes in volume in the foot or leg for swelling or changes in medications.

Risk mitigation: The principal investigator will ensure that the footwear and insole fit well on the participant’s foot without putting any pressure on the foot and leg. The footwear comes with removable spacer inlays, and the thickness of the insoles can be adjusted if needed. The participants will be given a written wearing information sheet with contact details in case of any emergency and advice to stop wearing them until having a review with any of the investigators.

iii. Risk of feeling discomfort or feeling unhappy about wearing a new kind of shoe and insole which may be quite different to what the participant is generally used to. Sometimes, participants may have perceptions that the orthopaedic or therapeutic footwear may not be aesthetically as appealing compared to their regular footwear.

Risk mitigation: The participant will be fully involved in and informed about the process of designing and manufacturing the prescribed footwear and insoles relating to their foot conditions during the first appointment. They will have input on design, style and colour selections for the footwear as per their intended activity. The footwear and insoles for the study will be used from premium orthopaedic brands and manufacturers to ensure the best possible quality and appearance.

### Frequency and plans for auditing trial conduct

This project is subjected to online audit tools of IRMA and REGIS for project progress, and project completion review is conducted yearly or before if the project is shorter than 12 months.

### Plans for communicating important protocol amendments to relevant parties

Any changes that become necessary during the study period will be proposed and amended in the study protocol and be made available to the HREC committee of NBMLHD and SCU. The research governance offices (RGOs) of each study site will also be notified through the Research Ethics Governance Information Systems (REGIS). SCU HREC will be reported through the Integrated Research Management Application (IRMA).

### Dissemination plans

Findings will be published in relevant scientific journals and presented at scientific meetings as well as in a PhD thesis.

## Discussion

A patient-centric study design for footwear intervention in people with diabetes is non-existent [40, 71]. National and international guidelines [35, 72] recommend footwear and insole features to prevent foot ulcer occurrence and recurrence in people with diabetes. They also highlight the importance of adherence to them for improved clinical outcomes, but the guideline on improved adherence is non-existent. Participant satisfaction and adherence to the prescribed footwear are vital to achieving the desired clinical outcome [40].

Footwear is an integral part of the wardrobe for every individual irrespective of gender and activity level. Consideration of participant expectations, geographical and socioeconomic factors, effective education on footwear and activity-specific device designs are important considerations for a personalised-treatment approach for increased adherence [40]. This proposed N-of-1 trial is set to bridge the gap in current clinical practice through evidence-based recommendations to improve offloading efficacy and adherence to the prescribed footwear and insoles in people with diabetes and neuropathy.

### Limitations

The study period is within the COVID-19 restriction in New South Wales, which may affect the study operation and the number of participant recruitment. It may be inconvenient for some potential participants. For example, hospital outpatient department visits require mandatory vaccination for eligible persons and a PCR test conducted within 72 h of the clinic visit.

## Trial status

The trial has been started in two study centres (Western Sydney Podiatry clinic since May 2021 and St Vincent’s Hospital since July 2021). Participant recruitment is expected to be completed by April 2022 in all study centres.

## Definition

Pedorthist: A person who provides medical-grade footwear and/or orthotic appliances and appropriate advice to a patient after assessment and analysis of the patient’s problem(s). This includes the provision of prefabricated footwear, modification of prefabricated footwear, custom-designed and manufactured footwear and/or orthotic appliances and advice on the need and application of medical-grade footwear, orthotic appliances and other footwear.

## Data Availability

The datasets during the study are available from the corresponding author on a reasonable request. All data will be de-identified before making it available to authorised parties.

## Abbreviations

ANOVA: Analysis of variance
CRF: Case report form
PIS: Participant information sheet
HREC: Human Research Ethics Committee
HRFS: High-risk foot service
IRMA: Integrated Research Management Application
NBMLHD: Nepean Blue Mountains Local Health District
NHMRC: National Health and Medical Research Council
PPP: Peak plantar pressure
QOLS: Quality of Life Scale
RDMP: Research Data Management Plan
RGO: Research governance office
SCU: Southern Cross University
